# Crystal Structure, Cytotoxicity and Interaction with DNA of Zinc (II) Complexes with o-Vanillin Schiff Base Ligands

**DOI:** 10.1371/journal.pone.0130922

**Published:** 2015-06-26

**Authors:** Mei-Ju Niu, Zhen Li, Guo-Liang Chang, Xiang-Jin Kong, Min Hong, Qing-fu Zhang

**Affiliations:** 1 School of Chemistry and Chemical Engineering, Liaocheng University, Liaocheng, Shandong, 252059, China; 2 Shandong Provincial Key Laboratory of Chemical Energy Storage and Novel Cell Technology, Liaocheng University, Liaocheng, Shandong, 252059, China; University of Edinburgh, UNITED KINGDOM

## Abstract

Two new zinc complexes, Zn(HL^1^)_2_ (**1**) and [Zn_2_(H_2_L^2^)(OAc)_2_]_2_ (**2**) [H_2_L^1^ = Schiff base derived from *o*-vanillin and (*R*)-(+)-2-amino-3-phenyl-1-propanol, H_3_L^2^ = Schiff base derived from *o*-vanillin and 2-amino-2-ethyl-1,3-propanediol], have been synthesized and characterized by single crystal X-ray diffraction, elemental analyses, TG analyses, solid fluorescence, IR, UV-Vis and circular dichroism spectra. The structural analysis shows that complex **1** has a right-handed double helical chain along the crystallographic b axis. A homochiral 3D supramolecular architecture has been further constructed by intermolecular C-H··· π, O-H···O and C-H···O interactions. Complex **2** includes two crystallographically independent binuclear zinc molecules. The two binuclear zinc molecules are isostructural. The 2-D sheet supramolecular structure was formed by intermolecular hydrogen bonding interaction. The fluorescence of ligands and complexes in DMF at room temperature are studied. The interactions of two complexes with calf thymus DNA (CT-DNA) are investigated using UV-Vis, CD and fluorescence spectroscopy. The results show that complex **1** exhibits higher interaction with CT-DNA than complex **2**. In addition, in vitro cytotoxicity of the complexes towards four kinds of cancerous cell lines (A549, HeLa, HL-60 and K562) were assayed by the MTT method. Investigations on the structures indicated that the chirality and nuclearity of zinc complexes play an important role on cytotoxic activity.

## Introduction

**Fig 1:**
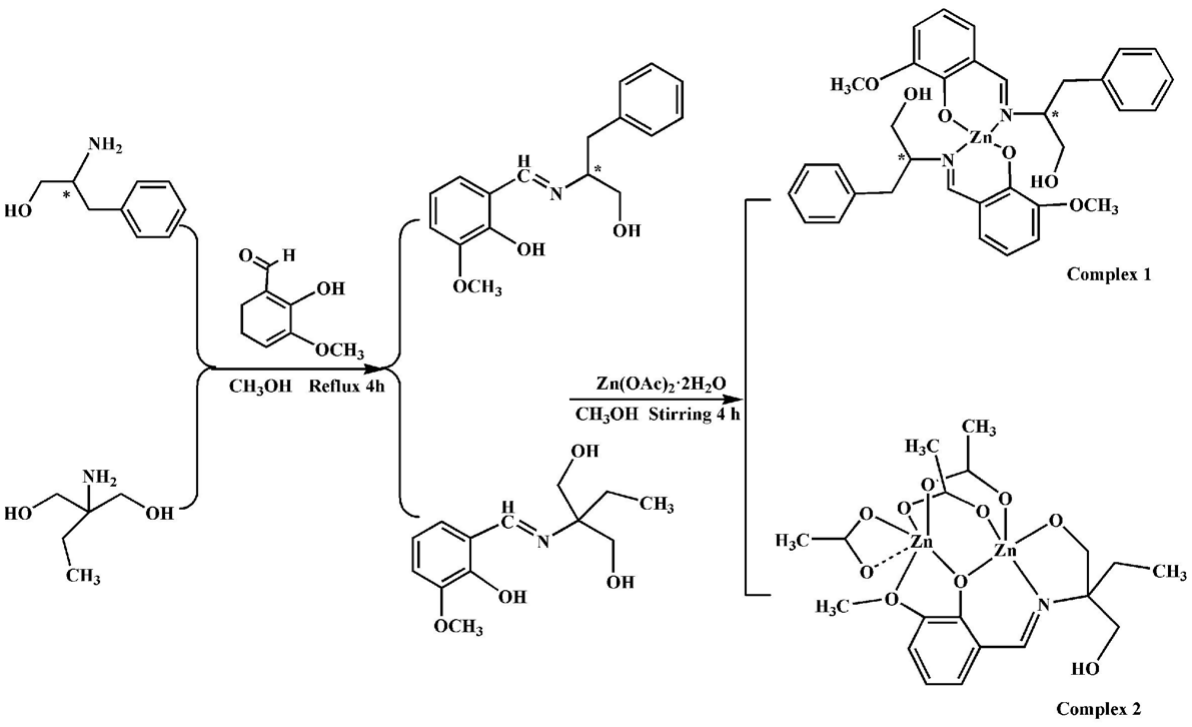
Syntheses of Schiff base ligands and complexes.

Schiff bases are considered as privileged and the most widely used ligands, due to their metal complexes having variety of applications in catalysis, functional materials, antibacterial, anticancer, optical resolution [1–5] and organic synthesis [6]. Thus, the rational design and synthesis of new Schiff base metal complexes are very meaningful. In recent years, Schiff base zinc complexes, especially chiral Zn (II) complexes have attracted considerable research interest owing to their various coordination modes and special properties. Until now researchers have explored their potential applications in luminescent materials [7,8], SHG–active materials [9], fluorescent sensor [10,11]. For instance, Consiglio et al. [12] reported the synthesis and fluorescence properties of Zn (II)–Schiff–base complexes with 2–hydroxy–4–(undec–10–enyloxy)benzaldehyde and 1,2–diamine derivates. Roy et al. [13] reported two hexanuclear zinc (II) complexes with Schiff base ligands and explained their fluorescence properties. To this class of compounds, their anticancer activities are also hot topics [14,15]. It is important to understand the DNA binding of complexes containing zinc (II) ions and their possible relationship to cytotoxicity in tumor cell lines [16,17]. Among the most accessed methods for investigating drug–DNA interactions, the procedure using fluorescent changes of Ethidium bromide (abbr. EB)-DNA system is attractive in nucleic acids chemistry owing to the high sensitivity and good accuracy [18]. Recently we have been focused on designing novel Schiff base polydentate ligands and investigating their self-assembly with metal centers, as well as their properties of corresponding complexes. We have reported several Schiff base nickel (II), cobalt (II)/(III) and copper (II) complexes, which exhibited significant effect for CT-DNA binding ability and cytotoxic activity [19–21]. To explore Schiff base zinc complexes as anticancer agents and stable blue fluorescent materials, two kinds of Schiff base zinc complexes were synthesized using *o*-vanillin as Schiff base ligands (Fig 1). Their interactions with CT-DNA were also investigated by using UV-Vis, circular dichroism and fluorescence spectra. The in vitro cytotoxic effect of these complexes on cancerous cell lines, including human lung carcinoma cell line (A549), chronic myelogenous leukemia cells line (K-562), human promyelocytic leukemia cells (HL-60) and human colon carcinoma cell lines (HCT-116), showed that chiral complex **1** exhibited substantial cytotoxic activity. In addition, complex **1** also exhibited significant effect for CT-DNA binding ability. The results indicated that the chirality and nuclearity have improtant influence on their anticancer activities and fluorescent emission.

## Experimental Section

### Materials and method

All chemicals are commercially available and used without further purification. IR spectra were recorded on Nicolet-5700 FT-IR spectrophotometer with KBr pellets in the 4000–400 cm^-1^ region. UV-Vis spectra were performed on a UV-2550 ultraviolet spectrophotometer. ^1^H and ^13^C NMR spectra were obtained on a Varian Mercury Plus 400 MHz NMR spectrometer. Elemental analyses for C, H and N were performed at a PE-2400-II apparatus. UV-vis absorption spectra were recorded on an HP-8453A diode array spectrophotometer. Fluorescence spectra were recorded on an LS55 spectrofluorometer. Circular dichroism (CD) spectra measurements were conducted on a Jasco J-810 spectropolarimeter. Thermal analyses were performed with a TGA SDTA 851e thermogravimetric analyzer. The Zn elemental contents for the complexes were measured by PE-Optima 2100 DV apparatus.

### Syntheses

#### Synthesis of H_2_L^1^

The ligands and complexes were synthesized following the procedure shown in Scheme 1. The methanol solution of (R)-(+)-2-amino-3-phenyl-1-propanol (0.151g, 1 mmol) was added to *o*-vanillin (0.152g, 1 mmol) in 10 mL methanol. The mixed solution was refluxed under stirring for 3–4 h. The resulting yellow solution was filtered and dried in vacuum. Then the yellow product of **H**_**2**_ **L** ^**1**^ was obtained, which was washed with ether for three times and dried at room temperature. Yield: 86.3%; M.p.: 130–131°C; *Anal*. Calc. for C_17_H_19_NO_3_ (%): C, 71.56; H, 6.71; N, 4.91. Found: C, 71.49; H, 6.65; N, 4.98. IR (KBr pellet: cm^-1^): 3431 (s, _O–H_), 2926(s, _C–H_), 1639 (s, _C = N_), 1248(w, _Ph–O_). ^1^H NMR (400 MHz, CDCl_3_) δ/ppm: 8.07 (–C**H** = N); 7.13–7.26 (Ph–**H**); 6.72–6.76 (Ph–**H**); 3.85 (–OC**H**_3_); 3.73–3.77 (–C**H**_2_OH); 3.52 (chiral–C**H**); 1.04 (Ph–C**H**_2_–). ^13^C NMR (400 MHz, CDCl_3_), δ/ppm: 166.25 (–**C**H = N); 113.96–151.92 (Ph–**C**); 72.94 (–**C**H_2_OH); 65.90 (chiral–**C**); 56.10 (–O**C**H_3_); 39.08 (–**C**H_2_–). UV/Vis [CH_3_CH_2_OH; λ_max_/nm]: 419, 324, 295. Specific optical rotation [α]λ^25^ = +287.5° (c, 1, ethanol). CD λ_max_ (Δε) (ethanol): 417 (+1.04), 328 (+2.24), 261 (+8.93).

#### Synthesis of H_3_L^2^

**H**_**3**_ **L** ^**2**^ was prepared by the similar procedure that given in the case of **H**_**2**_ **L** ^**1**^, using 2-amino-2-ethyl-1,3-propanediol (0.119g, 1 mmol) and *o*-vanillin (0.152g, 1 mmol) as reactants. The yellow product of **H**_**3**_ **L** ^**2**^ was obtained. Yield: 81.7%; M.p.: 149–151°C; *Anal*. Calc. for C_13_H_19_NO_4_ (%): C, 61.66; H, 7.51; N, 5.53. Found: C, 61.57; H, 5.46; N, 5.74. IR (KBr pellet: cm^-1^): 3430 (s, _O–H_), 2925(s, _C–H_), 1637 (s, _C = N_), 1221 (w, _Ph–O_). ^1^H NMR (400 MHz, CDCl_3_) δ/ppm: 8.58 (–C**H** = N); 6.97–7.22 (Ph–**H**); 3.64–3.93 (–C**H**_2_OH); 3.83 (–OC**H**_3_); 1.62 (–C**H**_2_–); 0.92(–C**H**_3_). ^13^C NMR (400 MHz, CDCl_3_), δ/ppm: 166.07 (–**C**H = N); 115.34–152.34 (Ph–**C**); 63.43 (–**C**H_2_OH); 56.32 (tert–**C**); 56.11 (–O**C**H_3_); 22.82 (–**C**H_2_–); 9.84 (–**C**H_3_). UV/Vis [CH_3_CH_2_OH; λ_max_/nm]: 432, 357, 298.

#### Synthesis of Complex 1

Zn(OAc)_2_·2H_2_O (0.110g, 0.5 mmol) in 5 mL methanol was slowly added to a stirred methanol solution (15 mL) of the **H**_**2**_ **L** ^**1**^ (0.285 g, 1 mmol), the yellow mixture was stirred at room temperature for 4 h. Followed by filtration, yellow crystal of complex **1** which is suitable for X-ray diffraction was obtained by slow evaporation of the solvent after one week. Yield: 72.0%; M.p.: 255–257°C; *Anal*. Calc. for C_34_H_36_N_2_O_6_Zn (%): C, 64.40; H, 5.72; N, 4.42; Zn, 9.83. Found: C, 64.27; H, 5.61; N, 4.56; Zn, 10.30. IR (KBr pellet: cm^-1^): 3406 (s, _O–H_), 2930 (m, _C–H_), 1628 (m, _C = N_), 1240 (w, _Ph–O_), 558 (m, _Zn–N_), 469 (m, _Zn–O_). ^1^H NMR (400 MHz, CDCl_3_) δ/ppm: 8.05 (–C**H** = N); 6.18–7.24 (Ph–**H**); 3.89 (–OC**H**_3_); 3.71–3.79 (–C**H**_2_OH); 3.46 (chiral–C**H**); 1.10 (Ph–C**H**_2_–). ^13^C NMR (400 MHz, CDCl_3_) δ/ppm: 166.05 (**C** = N); 114.34–152.34 (Ph–**C**); 70.25 (–**C**H_2_OH); 63.07(chiral–**C**); 56.19 (–O**C**H_3_); 34.81 (–**C**H_2_–). UV/Vis [CH_3_CH_2_OH; λ_max_/nm]: 375, 277, 243. Specific optical rotation [α]λ^25^ = +445.0° (c, 1, ethanol). CD λ_max_ (Δε) (CH_3_CH_2_OH): 362 (+7.13), 278 (+17.96), 243 (+8.37).

#### Synthesis of Complex 2

Zn(OAc)_2_·2H_2_O (0.219 g, 1 mmol) in 10 mL methanol was added dropwise to a stirring methanol solution (10 mL) containing **H**_**3**_ **L** ^**2**^ (0.2533 g, 1 mmol). The mixture was stirred for 4 h at room temperature and then filtered, yellow needle-like shaped crystal of complex **2** was obtained by slow evaporation of the methanol solution after two weeks. Yield: 63.5%; M.p.: 269–271°C; *Anal*. Calc. for C_19_H_27_Zn_2_NO_10_ (%): C, 40.73; H, 4.86; N, 2.50; Zn, 23.02. Found: C, 40.59; H, 4.91; N, 2.61; Zn, 23.34. Selected IR (KBr pellet: cm^-1^): 3433 (s, _O-H_), 2923 (m, _C–H_), 1632 (m, _C = N_), 1600 (s, as _COO_^-^), 1435, 1416 (s, s _COO_^-^), 1171 (m, _Ph-O_), 507 (m, _Zn-N_), 468 (m, _Zn-O_). ^1^H NMR (400 MHz, CDCl_3_) δ/ppm: 8.47 (–C**H** = N); 6.56–7.41 (Ph–**H**); 3.52–3.75 (–C**H**_2_OH); 3.81 (–OC**H**_3_); 1.67 (–C**H**_2_–); 1.22 (–C**H**_3_). ^13^C NMR (400 MHz, CDCl_3_), δ/ppm: 165.87 (–**C**H = N); 116.26–152.71 (Ph–**C**); 63.82 (–**C**H_2_OH); 55.94 (tert–**C**); 54.47 (–O**C**H_3_); 23.88 (–**C**H_2_–); 9.65 (–**C**H_3_). UV/Vis [CH_3_CH_2_OH; λ_max_/nm]: 390, 324, 283.

### X-ray Crystallography

Single-crystal X-ray diffraction datas were collected on a Bruker Smart-1000 CCD diffractometer equipped with graphite-monochromated Mo Kα (λ = 0.71073 Å) radiation at room temperature. The structures were solved by direct methods and refined by the full-matrix least-squares methods on *F* ^*2*^ using the SHELX-97 program. All non-hydrogen atoms were refined with anisotropic displacement parameters, while the hydrogen atoms were placed in calculated positions with isotropic displacement parameters and 1.2×*U*_*eq*_ of the attached atom. Crystal and refinement datas are summarized in Table 1. Selected bond lengths and angles are also listed in Table 1.

**Table 1:** Crystal and structure refinement data for complexes 1 and 2.

Complex	1	2
Empirical formula	C_34_H_36_N_2_O_6_Zn	C_19_H_27_NO_10_Zn_2_
Formula weight	634.02	560.16
Temperature	298(2) K	298(2) K
Crystal system	Monoclinic	Triclinic
Space group	*P*2_1_	*P*-1
*a/*nm	14.1396(12) Å	8.9725(8)
*b*/nm	7.2577(7) Å	10.0677(9)
*c*/nm	15.5893(15) Å	26.232(2)
*α*/(°)	90	89.632(2)
*β*/(°)	96.518(10)	89.835(2)
*γ*/(°)	90	68.6590(10)
*V*/nm^3^	1589.4(3) Å^3^	2.207(3)
*Z*	2	4
*D_c_*/(g•cm^-3^)	1.316	1.686
*F*(000)	664	1152
Reflrctions collected	8237	11264
*R*_int_	0.0290	0.0389
*R*_1_ [I>2σ(*I*)]^[a]^	0.0463	0.1502
*R*_1_ (all data)^[a]^	0.0588	0.1780
*wR*_2_ [I>2σ(*I*)]	0.1123	0.3235
*wR*_2_ (all data)	0.1193	0.3356
residual electron density	0.42/-0.35	1.18/-1.17
Flack	0.012(16)	

### Cytotoxicity

In vitro cytotoxicities of the complexes were studied using standard MTT assay bioassay in different cancer cells for 24 h of drug administration. The tested complexes were prepared for the experiment by dissolving in 0.1% DMSO and diluted with medium. Cell lines of A549, HCT-116, HL-60 and K-562 were cultured in 96-well culture plate in RPMI-1640 medium containing 10% FBS and 1% antibiotics, maintain culture at 37°C, 5% CO_2_ and 95% air in the CO_2_ incubator for 24 h. Different concentrations of prepared complexes were added to the cells and the incubation continued for 24 h. Then the media was removed, MTT was dissolved in medium and added to each well, then incubated for another 4 h. The purple formazan crystals were solubilized by the addition of 100 μL DMSO. A reading was taken on a plate reader, the absorbance was measured at 570 nm by the ELISA reader after the plate was shaken for 5 min. The values are the averages from at least three independent experiments, which were measured as the percentage ratio of the absorbance of the treated cells to the untreated controls. The IC_50_ values were determined by non-linear regression analysis.

### DNA-binding studies

UV-Vis absorbances was performed by keeping the concentration of the Zn (II) complexes (10 μM) constant while varying the CT-DNA concentrations from 0 to 10 μM. The sample solution was scanned in the range of 200–500 nm, while making the CT-DNA concentrations changed from 0 to 10 μM. For the fluorescence quenching experiment, the sample was added to the solution containing 30 μM CT-DNA and 3 μM EB at different concentrations (0, 15, 30, 60, 90, 120 μM). After 2 h, the fluorescence quenching spectra were recorded at 500−700 nm at *λ*_ex_ = 258 nm in the absence and presence of the complex. The spectrum was recorded at the scan speed of 150 nm/min both excitation and emission with slit width 10.0/10.0 nm. CD spectra of CT-DNA were carried out in the absence and presence of the complexes at the room temperature with a quartz cell of 1 cm path length. Each sample solution was scanned in the range of 220–320 nm with a scan speed of 100 nm/ min and 1 s response time. Each spectrum was the average of three accumulations from which the buffer background had been subtracted.

## Results and Discussion

### Spectra

#### Infrared spectra

The IR spectra of complexes **1** and **2** show strong band at 1628–1632 cm^-1^ assigned to the υ(C = N) vibration. Compared to the spectra of free ligand [υ(C = N) 1639–1637 cm^-1^], it shifts lower frequency by 5–11 cm^-1^ and supports the coordination of the υ(C = N) group [22]. The υ(Ph–O) vibration of free ligand (1221–1248 cm^-1^) locates at lower frequency for complexes (1171–1240 cm^-1^), it could be considered that the deprotonated phenol O group has coordinated to the Zn (II) ion [23,24]. Complex **1** has two intense absorption bands at 1600, 1435 and 1416 cm^-1^, may be attributed to the anti-symmetric and symmetric vibrations of the carboxylate groups. The appearance of 558–507 and 470–469 cm^-1^ are assigned to Zn–O and Zn–N absorption respectively [25,26].

#### UV-Vis spectra

The UV-Vis spectra of the ligands and complexes are recorded in ethanol. The ligands showed three main bands in the range 295–298 nm, 324–357 nm and 419–432 nm, respectively, which may be attributed to π–π* and n–π* transitions of the aromatic rings and the non-bonding electrons on N atoms of imino groups [27]. While complexes display three strong absorption bands at 243–283 nm, 277–324 nm and 375–390 nm, largely blue–shifted as compared to the bands of the ligands. It does not exhibit d–d transition due to its completely filled d^10^ electronic configuration [28].

#### Circular dichroism spectra

**Fig 2:**
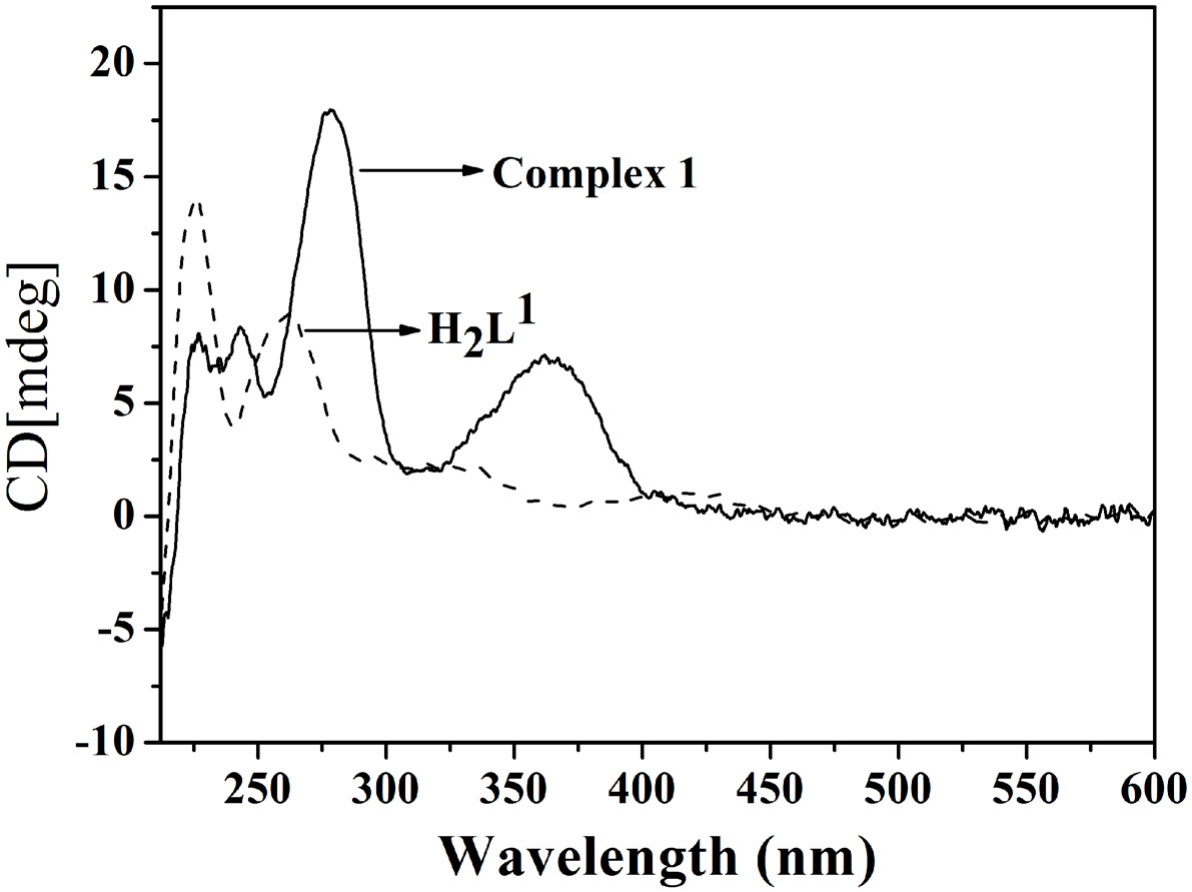
Circular dichroism spectra of the H_2_L^1^ and complex 1.

Circular dichroism spectra have been utilized as a powerful tool for exploring the chiral aspect of complexes [29]. The CD spectra of **H**_**2**_ **L** ^**1**^ and complex **1** in the buffer solution are shown in Fig 2. The CD spectrum of **H**_**2**_ **L** ^**1**^ shows three positive bands (ILCT) around 417, 328 and 261 nm. These peaks correspond to the UV-vis absorption peaks observed around 419 and 324 nm. The CD spectrum of complex **1** also shows three peaks, but at shifted positions corresponding to the UV transitions at 362, 278 and 243 nm. Complex **1** and **H**_**2**_ **L** ^**1**^ have been tested both as dextroisomer and their specific optical rotation are [α]λ^25^ = +445.0°, [α]λ^25^ = +287.5°.

Investigations on the circular dichroism spectroscopy indicate that synthetic **H**_**2**_ **L** ^**1**^ and complex **1** are chiral compounds.

### Thermal analysis

The thermogram of the complexes **1** and **2** were recorded under nitrogen atmosphere at the heating rate of 10°C/min (S1 Fig). Before 260°C, TG curves of complex **1** show that the thermogram does not display any inflexion point, which indicates that complex **1** has excellent thermal reliability, and it does not contain any kind of small ligands (water or acetic acid). Upon heating above 260°C, a rapid collapse took place, indicating the decomposition of the complex **1**. The final residue may be a mixture of ZnO and carbon. The TG curve of complex **2** shows two stages. In the range of 220–320°C, the TG curve shows the first gradual weight loss, corresponding to the loss of the coordinated acetic acid. In the range of 330–800°C, there is a quick weight loss, which indicates that the Schiff base ligands begin to rapidly decompose. The final residue of complex **2** is similar to complex **1**.

### Description of crystal structure

#### Complex 1

**Fig 3:**
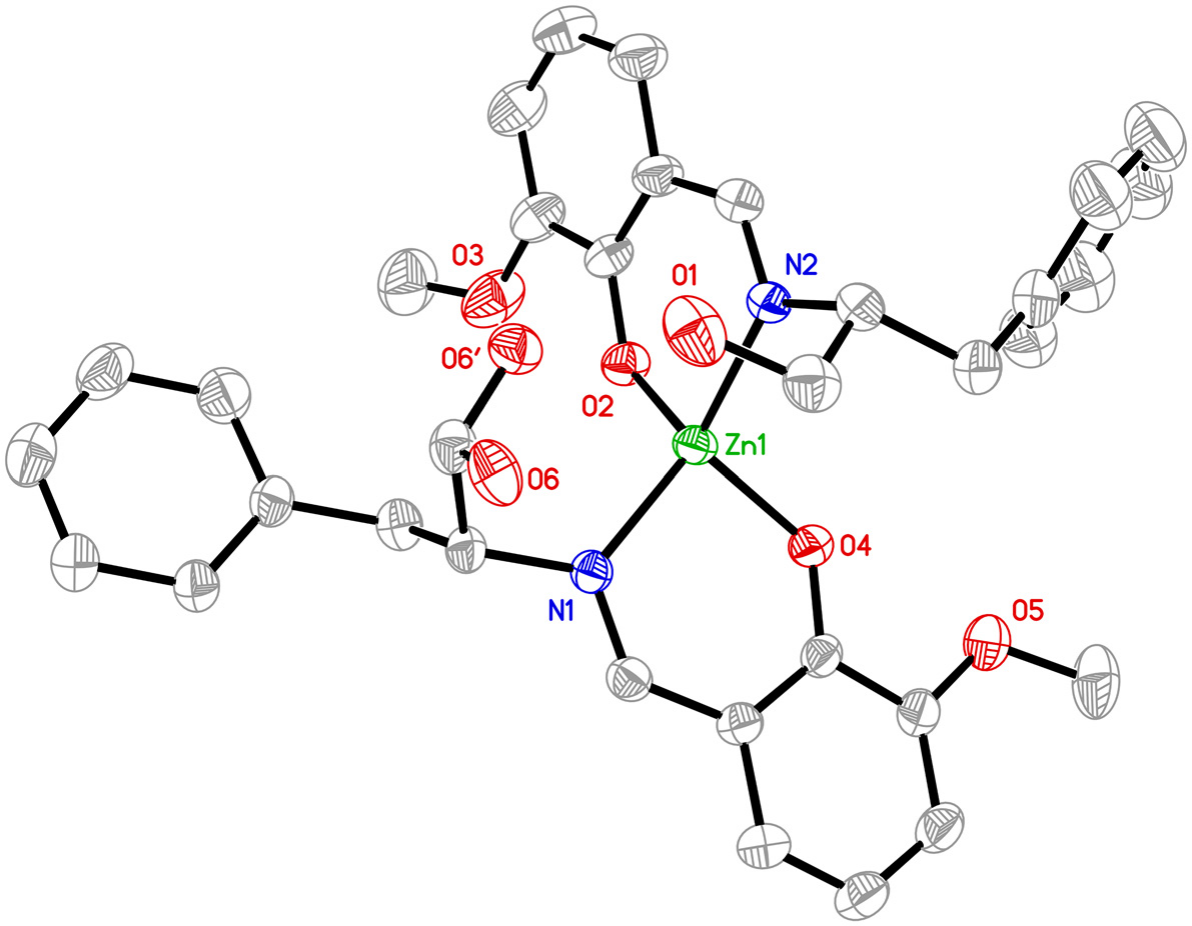
Molecular structures of complex 1, hydrogen atoms are omitted for clarity (Thermal ellipsoids are shown at 50% probability level).

Single crystal X-ray diffraction analysis reveals that complex **1** crystallizes in monoclinic space group *P*2_1_, and consists of one Zn (II) ion and two chiral HL^1-^ in the asymmetric unit. As shown in Fig 3, the central Zn (II) ion is four-coordinated by two deprotonated phenolic O atoms and two imino N atoms from two different chiral HL^1-^ ligands, displaying a distorted tetrahedral geometry with six bond angles ranging from 95.97(15) to 129.70(19°. The Zn–O and Zn–N bond distances are in the normal range of Zn-Schiff base complexes [30]. The two chiral HL^1-^ ligands in complex **1** are both mono-deprotonated, namely, only the phenolic–OH group is deprotonated, which is consistent with the IR spectrum analysis (vide supra). Therefore, the two chiral HL^1-^ ligands exhibit a bidentate N:O chelate mode to bind the central Zn (II) ions and form two stable six-member ring structures [C(1)–C(2)–C(3)–N(1)–O(4)–Zn(1); C(18)–C(19)–C(20)–N(2)–O(2)–Zn(1)], which are arranged in a closely perpendicular mode (the dihedral angle of C(1)–C(2)–C(3)–N(1)–O(4)–Zn(1) and C(18)–C(19)–C(20)–N(2)–O(2)–Zn(1) is about of 81.20°) between each other in order to satisfy the steric requirement of the two larger HL^1-^ ligands. The detailed structural analysis shows that the configurations of the chiral carbon atoms [C(10) and C(27)] in complex **1** are (*R)*-modes, which suggests that no chiral inversion of the HL^1-^ ligand has been observed and the chirality of the H_2_L^1^ free ligand has been successfully transferred into the Zn-complex.

**Fig 4:**
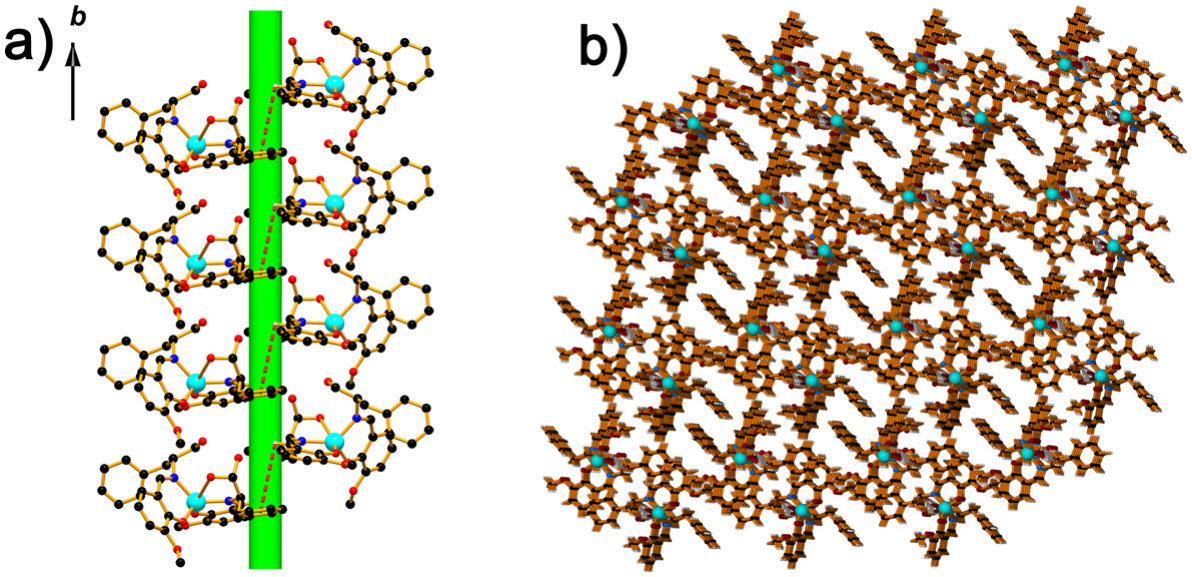
**(a)** Right-handed twofold helical chain in 1 linked by intermolecular C–H···π interactions (red dotted line); **(b)** 3D supramolecular architecture construcated by C–H···π, O–H···O and C–H···O interactions.

As shown in Fig 4a, the mononuclear molecules of complex **1** are connected by intermolecular C–H···π weak interaction (C1–H1A···π#1, d[C1–H1A] = 0.93 Å, d[H1A···π#1] = 3.84 Å, d[C1···π#1] = 4.146 Å, ∠[C1–H1A···π#1] = 102.8°, symmetric operation code: #1, 1–x, y+1/2, 1–z) to generate a right-handed twofold helical chain along the *b* direction with a pitch about of 7.258 Å. In addition, the O–H···O interactions (O1–H1···O2#1, d[O1–H1] = 0.82 Å, d[H1–O2#2] = 2.01 Å, d[O1···O2#2] = 2.698(6) Å, ∠[O1–H1···O2#2] = 141.4°, and O6–H6···O1, d[O6–H6] = 0.82 Å, d[H6–O1] = 1.74 Å, d[O6···O1] = 2.559(7) Å, ∠[O6–H6···O1] = 174.4°) are observed in the helical chain, which are believed to consolidate the helical chain structure. Furthermore, the intermolecular C–H···O weak interactions (C13–H13···O6#1, d[C13–H13] = 0.93 Å, d[H13···O6#1] = 2.67 Å, d[C13···O6#1] = 3.573(8) Å, ∠[C13–H13···O6#1] = 163.1°; C26 –H26B···O4#2, d[C26–H26B] = 0.97 Å, d[H26B···O4#2] = 2.52 Å, d[C26···O4#2] = 3.434(7) Å, ∠[C26–H26B···O4#2] = 157.6°; symmetric operation codes: #1, 1-x, y+1/2, 1-z; #2, x, y+1, z) are found to help to construct a homochiral 3D supramolecular architecture (Fig 4b), all the C–H···π, O–H···O and C–H···O interactions observed in complex **1** could be considered as the key factor to transmit chiral information form single molecules to the homochiral 3D supramolecular architecture and yield the chiral crystals finally.

#### Complex 2

**Fig 5:**
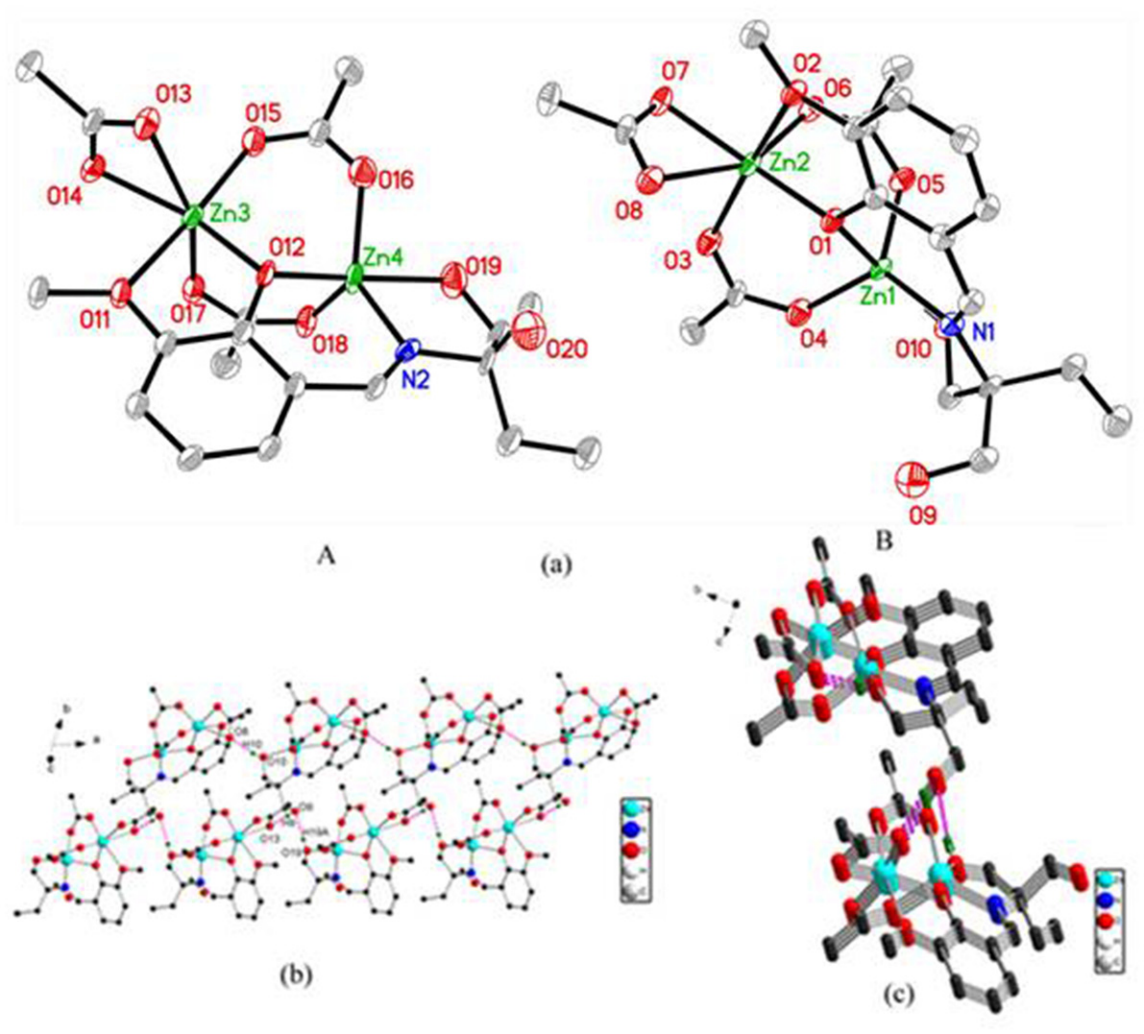
**(a)** Molecular structures of complex 2, hydrogen atoms are omitted for clarity (Thermal ellipsoids are shown at 50% probability level.); **(b)** View showing the 3–D structure of complex 2; **(c)** The independent units A and B appear in pair connecting the infinite chain.

Complex **2** crystallizes in space group *P*-1 and includes two crystallographically independent binuclear zinc molecules (denoted **A** and **B**, respectively) (Fig 5a). The two binuclear zinc molecules are isostructural and only molecule **A** is described representatively. In molecule **A**, the Zn1 center is five-coordinated with the ZnNO_4_ donor set in a distorted trigonal bipyramidal geometry (τ = 0.685). The equatorial plane are occupied by one imine nitrogen atom (N1) and two carboxylate oxygen atoms (O4, O5) from two bis(monodentate) bridging carboxylate groups, while one phenolic oxygen atom (O1) and one protonated alkoxo oxygen atom (O10) are positioned at the apical positions with the O1–Zn1–O10 angle of 165.1(4°. The deviation of Zn1 center from the least-squares plane defined by the three equatorial donor atoms is 0.1686(64) Å in **A** (while 0.9793(73) Å for Zn4 ion in **B**).

The Zn2 ion is five-coordinated with three oxygen atoms (O3, O6 and O7) from three carboxylate groups, one phenolic oxygen atom (O1) and one methoxy oxygen atom (O2), forming a slightly distorted square pyramid ZnO_5_ (τ = 0.16) with the O6 atom at the apical position. The bond angles from the apical atom to the basal atoms are in the range of 94.0(3)–100.8(4° for Zn2 in **A** (94.7(4)–98.7(4° for Zn3 in **B**), indicating the distortion from the ideal square pyramid. The deviation of Zn2 ion from the best plane formed by the basal donor atoms is 0.2849(45) Å in **A** (while 0.2554(47) Å for Zn3 ion in **B**). The Zn(1),·Zn(2) and Zn(3),·Zn(4) distances are 3.208(2) and 3.189(2) Å respectively.

The molecule **A** connects with each other in the **AA** sequence through the weak intermolecular hydrogen bonds O10–H10···O8 (symmetry code: x–1, y, z), forming a 1–D infinite chain. The independent moiety **B**, which appears in pairs with the moiety **A**, links with the infinite chain through the strong hydrogen-bonding interactions O9–H9···O13^i^ (symmetry code: i, x–1, y–1, z) and O19–H19A···O9^ii^ (symmetry code: ii, x, y+1, z), forming a 2–D sheet supramolecular structure (Fig 5b).

### Photoluminescence property

**Fig 6:**
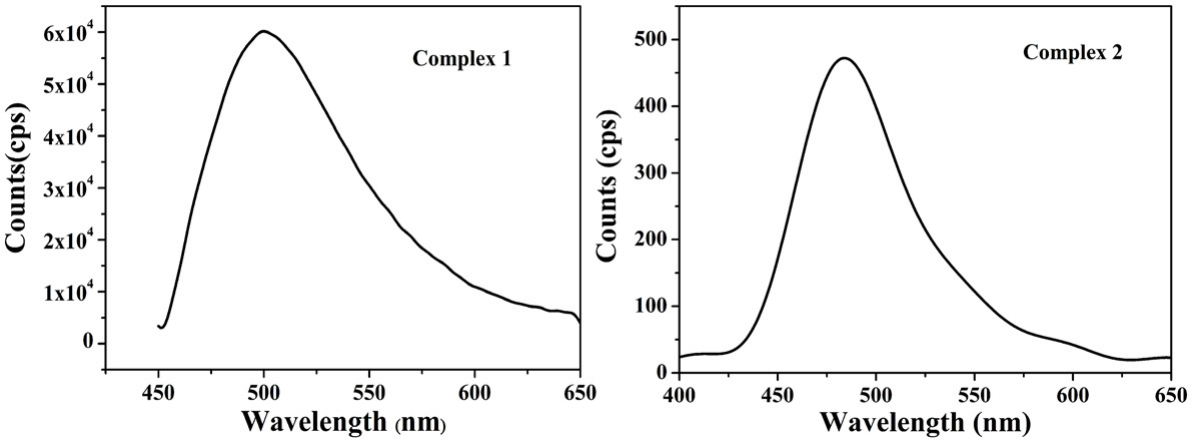
Solid–state emission spectra of complexes 1 and 2 at room temperature.

The photoluminescence (PL) property of the ligands and complexes are studied in the solid–state at room temperature as shown in Fig 6, S2 and S3 Figs. The free ligand H_2_L^1^ displays a luminescence with an emission maximum at 517 nm excited at 440 nm, and complex **1** shows a similar strong luminescence with emission maximum peaks at 502 nm (with λ_ex_ = 440 nm). The complex **2** exhibits weak emission at 483 nm upon excitation at 352 nm, relative to that of the corresponding ligand H_3_L^2^ (λ_em_ = 514 nm, λ_ex_ = 468 nm) and complex **1**. These emissions are neither metal-to-ligand charge transfer (MLCT) nor ligand-to-metal transfer (LMCT) in nature since the full filled d^10^ configuration of Zn^2+^ ions, which can probably be assigned to the intraligand (π-π*) fluorescent emission of benzene [31]. Investigations on the photoluminescence (PL) property shows the intensity of complex **1** is better than complex **2**, which is probably due to the differences of ligands and coordination environment around central Zn (II) ions [32]. Thus, the high luminescence efficiency in the blue light region and the thermally stability indicates the chiral complex **1** may be excellent candidate of stable blue fluorescent materials.

### In Vitro Cytotoxic Activity Evaluation of the complexes

**Fig 7:**
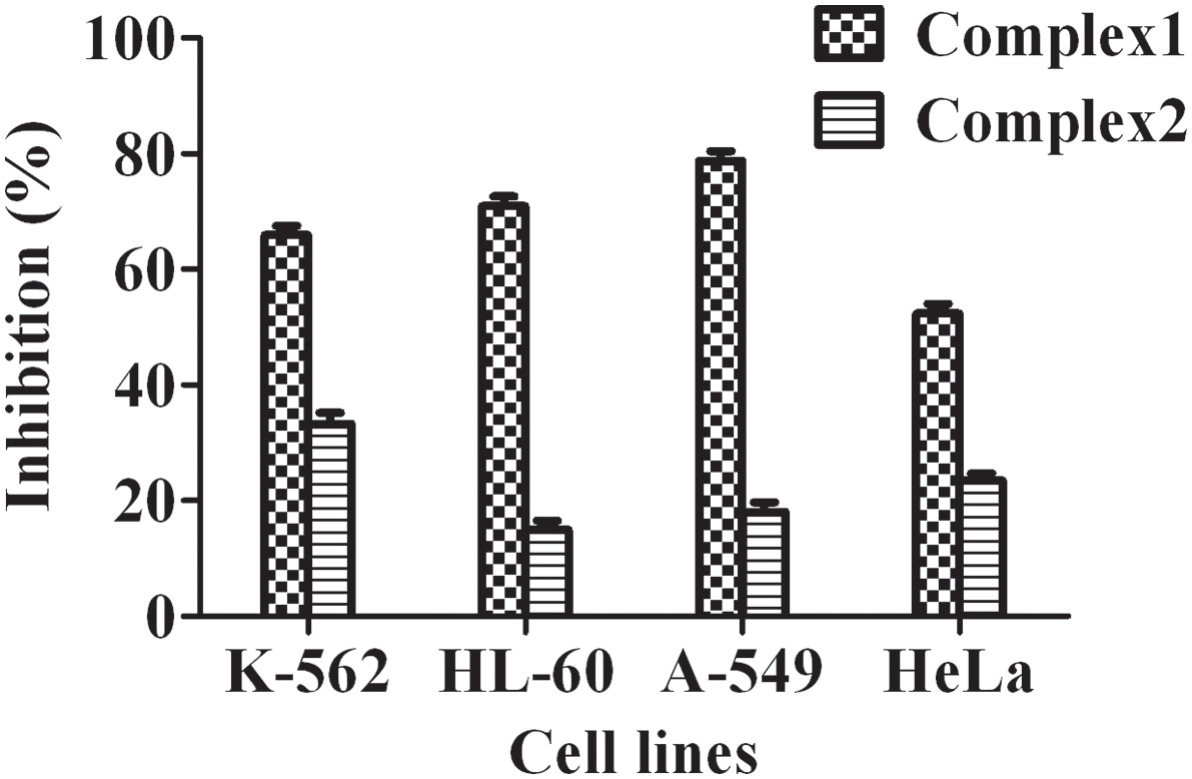
Inhibition [%] of complexes 1 and 2 [dose level of 25.0 μM] against human tumor cells.

In vitro cell culture studies are valuable tools for the screening of chemotherapy agents and provide preliminary datas for further studies. The cytotoxicities of ligands, complexes and zinc acetate to different cells were evaluated through the loss of cell viability using MTT assay. The inhibition effects of complexes **1** and **2** against the four cell lines at a concentration of 25.0 μM are listed in Fig 7. The IC_50_ values against four cell lines K-562, HL-60, A-549 and Hela are shown in Table 2. Generally, most physical, chemical and biological functions of therapeutic agents are strongly dependent on special structure of compounds. In contrast to DDP and CBP, complex **1** displays novel chiral structures. The experimental results indicated that the cytotoxicities are significantly dependent on the structures of the zine complexes, including the chiral and the nuclearity. The mononuclear chiral complex **1** is most significantly remarkable on all tested four cell lines, especially HL-60 and A549. Moreover, complex 1 was found to be more potent than cisplatin. The special chiral structure of complex **1** is probably contributed to their diverse bio-functions on tumor proliferation and causes a conformational change on DNA.

**Table 2:** IC_50_ (μM) of all complexes against K-562, HL-60, A549, and HeLa for 48 h treatment.

Complex	K-562	HL-60	A549	HeLa
**1**	27.27±1.36	17.75±1.14	15.60±1.12	24.18±1.14
**2**	44.58±1.25	>50	>50	>50
H_2_L^1^	>50	>50	>50	>50
H_3_L^2^	>50	>50	>50	>50
[Zn(OA_C_)_2_]·2H_2_O	>50	>50	>50	>50
Cisplatin	>50	>50	>50	>50

### DNA binding studies

#### Competitive binding between EB and complexes for CT-DNA

**Fig 8:**
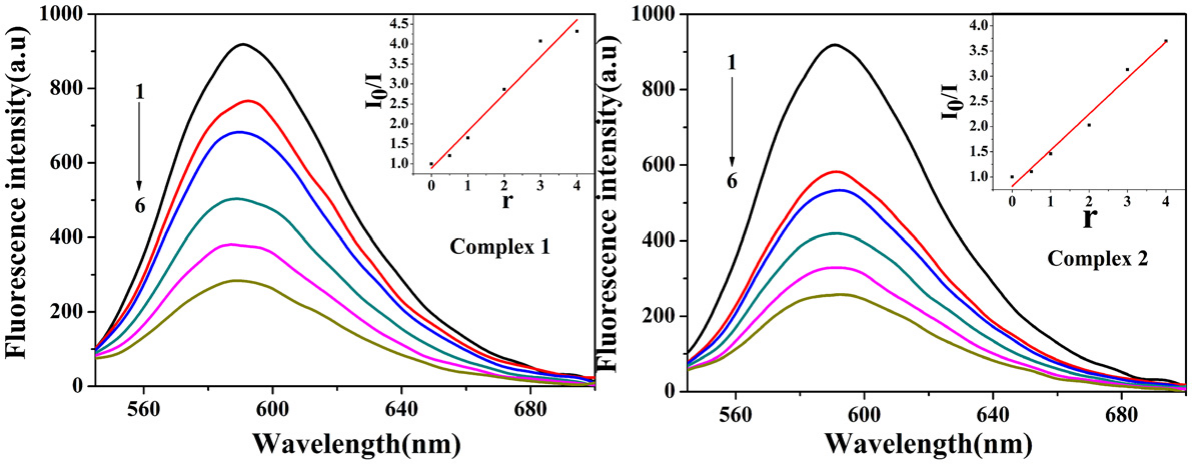
Effects of complexes 1 and 2 on the fluorescent spectra of EB–DNA system (λex = 258 nm); CDNA = 30 μM; CEB = 3 μM; from 1 to 6 C_VOL_ = 0, 15, 30, 60, 90, 120 μM, respectively; Inset: plot of *I*_0_/*I* vs *r* (r = C_VOL_/C_DNA_).

The competitive binding experiments using metal complexes as quenchers could provide some information about the binding of the complexes to DNA. This displacement method serves as an indirect evidence to identify intercalative binding modes. In this work, we studied that the interaction of two Zn (II) complexes with DNA was evaluated by the EB–DNA adduct, which can be used to distinguish intercalating and non intercalating compounds [18]. Competitive binding of other intercalators leads to a loss of fluorescence because of depletion of the EB–DNA complexes. The emission spectra of EB–DNA system in the presence and absence of complex **1** and **2** are shown in Fig 8. Fluorescence emission intensity of DNA–EB system are remarkably quenched at 590 nm as the concentration of complexes increase stepwise, this maybe because Zn (II) complexes could replace of EB and binds to DNA in an intercalative mode in the strong degree. The plot of *I*_o_/*I* versus *r* [complex]/[DNA] is inserted. The Stern-Volmer quenching constant, *K*_sq_, for the complexes **1** and **2** were 0.94, 0.75, respectively. These values are higher than those for some other complexes (*K*_sq_ = 0.73 and 0.52) [33], and the intensity of chiral complex **1** is better than achiral complex **2**.

### UV-Vis absorption studies

**Fig 9:**
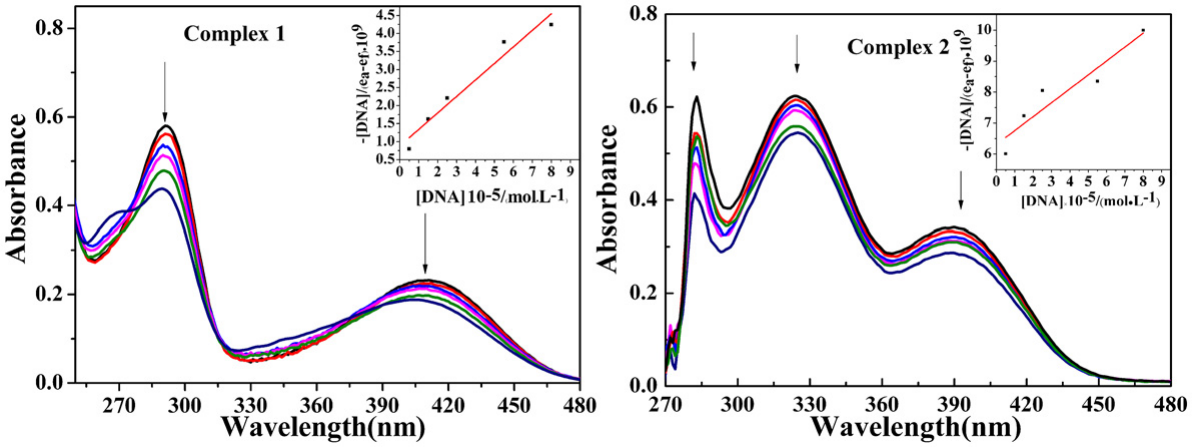
UV-Vis absorption spectra of complexes 1 and 2 (10 μM) in the absence and presence of increasing amounts of DNA (0–10 μM). Arrow shows the absorbance changes upon increasing DNA concentration.

To determine the binding ability of Zn (II) complexes with DNA helix, electronic absorption spectroscopy is employed [2]. When stacking interaction occurred between the aromatic chromophore of the test complexes and DNA base pairs, hypochromism and bathochromic shift in wavelength could be observed. The extent of hypochromism is commonly consistent with the strength of intercalative interaction [34]. The absorption spectrum of complexes **1** and **2** in the absence and presence of CT-DNA are shown in Fig 9. The strong absorption spectra at about 360 nm for tow complexes may be assigned to the ligand-to-metal charge transfer (LMCT). With the increase of the concentration of DNA, all the adsorption bands showed hypochromism accompanied with bathochromic shifts, similar to those previously reported metal intercalators. Generally, the complex could intercalate to the base pairs of DNA. And the π* orbital of the intercalators may couple with the π orbital of the base pairs, decrease the π-π* transition probabilities and consequently lead to hypochromism. The intrinsic binding constant *K*_b_ was calculated according to the equation: [DNA]/(*ε*_*a*_−*ε*_*f*_) = [DNA]/(*ε*_*b*_−*ε*_*f*_) + 1/*K*_b_(*ε*_a_−*ε*_f_) [35]. The K_b_ values for complex **1**−**2** were calculated to be 5.21×10^4^, 7.11×10^3^ M^-1^, respectively. From the results of the binding constants, the intrinsic binding constant *K*_b_ values of chiral complex **1** are higher than achiral complex **2**. This is consistent with the experimental results of fluorescence quenching.

### CD spectroscopy studies

**Fig 10:**
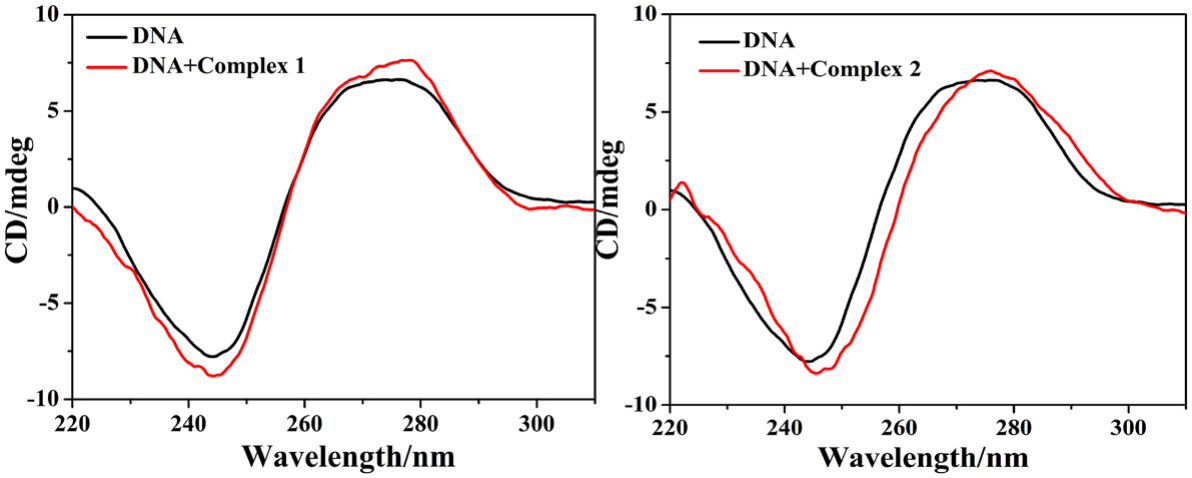
CD-spectra of CT-DNA in the absence and presence of complexes 1 and 2, [DNA] = 100 μM, [VOL] = 0 and 40 μM, respectively.

CD spectroscopy is one of the most sensitive ways and used to elucidate the changes, the conformational variations of DNA upon interaction with small molecules (often organic ligands or metal complexes) in solution. The CD spectra of CT-DNA on the addition of complexes **1** and **2**, exhibit significant perturbations both in negative and positive bands (shown in Fig 10). CD spectrum of CT-DNA exhibits a positive band at 274 nm (base stacking) and a negative band at 244 nm (helicity). The observed CD spectrum of CT DNA consists of a positive band at 274 nm due to base stacking and a negative band at 244 nm caused by helicity, which are characteristic of DNA in right-handed B-form. However, the positive and negative bands of complexes **1, 2** exhibited increase in intensity with a slight red-shift of the maximum which was attributed to partial insertion of planar aromatic chromophores in between the DNA base pairs causing stabilization of base stacking consequently [2]. The CD spectra also showed that the binding of the complex to CT-DNA does not lead to a significant change in the helix conformation of CT-DNA.

## Conclusion

The novel chiral and achiral Zn (II) complexes based on *o*-vanillin Schiff base ligand had been synthesized and structurally characterized. The structural analysis exhibited that a homochiral 3–D supramolecular architecture complex **1** has been further constructed by intermolecular C–H···π, O–H···O and C–H···O interactions. The chirality of complex **1** was further confirmed by CD spectra. Complex **2** includes two crystalographically independent binuclear zinc molecules, and the 2–D sheet supramolecular structure was formed by intermolecular hydrogen bonding interactions. Their interactions with CT-DNA were also investigated using UV-visible, fluorescence and synchronous fluorescence spectroscopic methods. The results showed chiral complex exhibited more efficient DNA interaction with respect to achiral. Also, the chiral Zn (II) complex shows more potent cytotoxic activity on selected cancerous cell lines. Therefore, the chiral and nuclearity of zinc complexes have large influence on the anticancer activities and fluorescent emission.

## Supporting Information

S1 Fig TG curve of the complex 1 and 2. (DOCX)

S2 Fig The solid–state emission spectra of H_2_L^1^ ligand at room temperature. (DOCX)

S3 Fig The solid–state emission spectra of H_3_L^2^ ligand at room temperature. (DOCX)

S1 Table Selected bond lengths (Å) and bond angles (°) for complexes 1 and 2. (DOCX)
