# Supplementary figures and images for: Crystal Structure, Cytotoxicity and Interaction with DNA of Zinc (II) Complexes with o-Vanillin Schiff Base Ligands
Source: PLoS One. 2015 Jun 26;10(6):e0130922. doi: 10.1371/journal.pone.0130922 (PMC4482705; doi:10.1371/journal.pone.0130922)

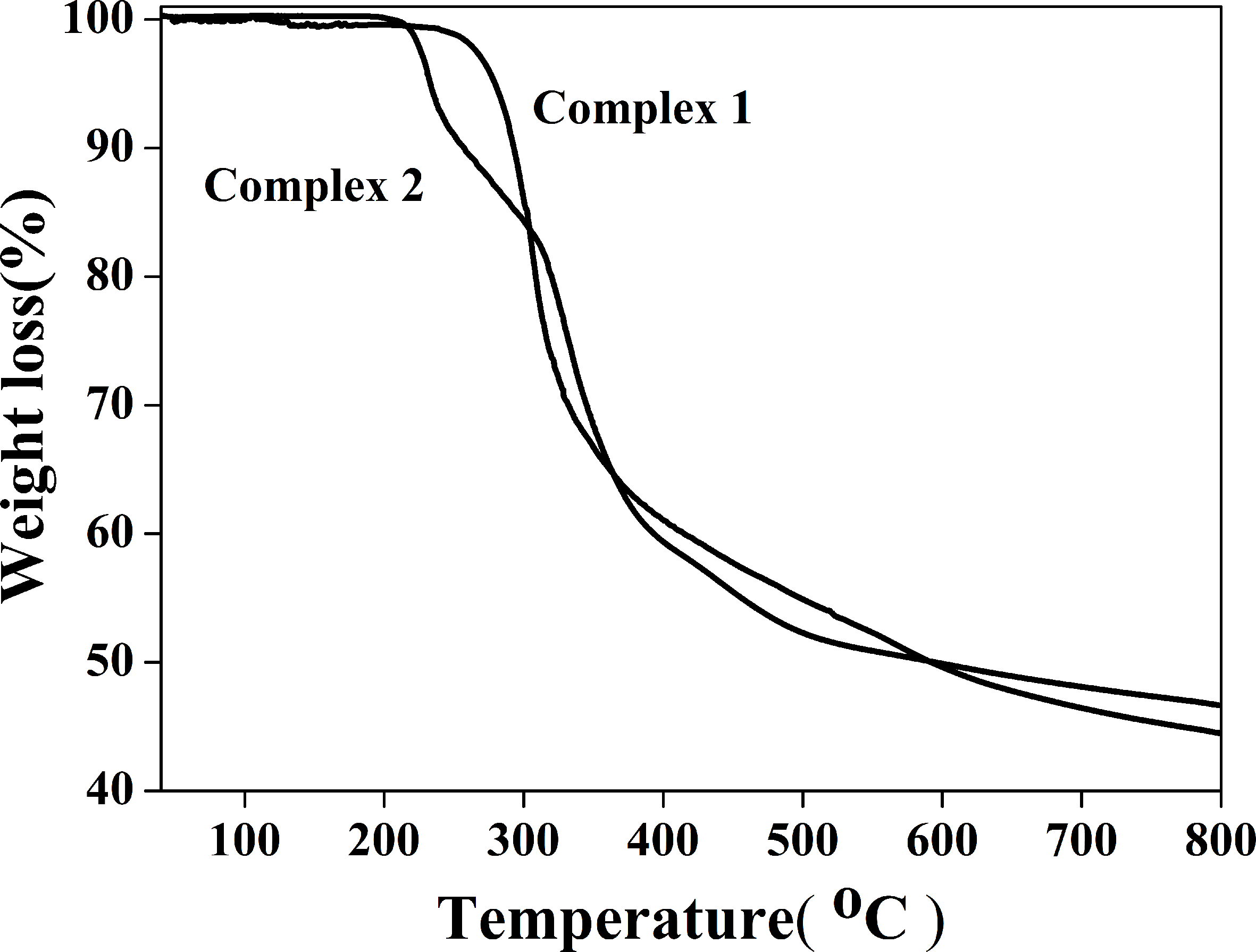


**S1 Fig. TG curve of the complex 1 and 2.**

Supplement: S1 Fig — (DOCX) [file pone.0130922.s001.docx]

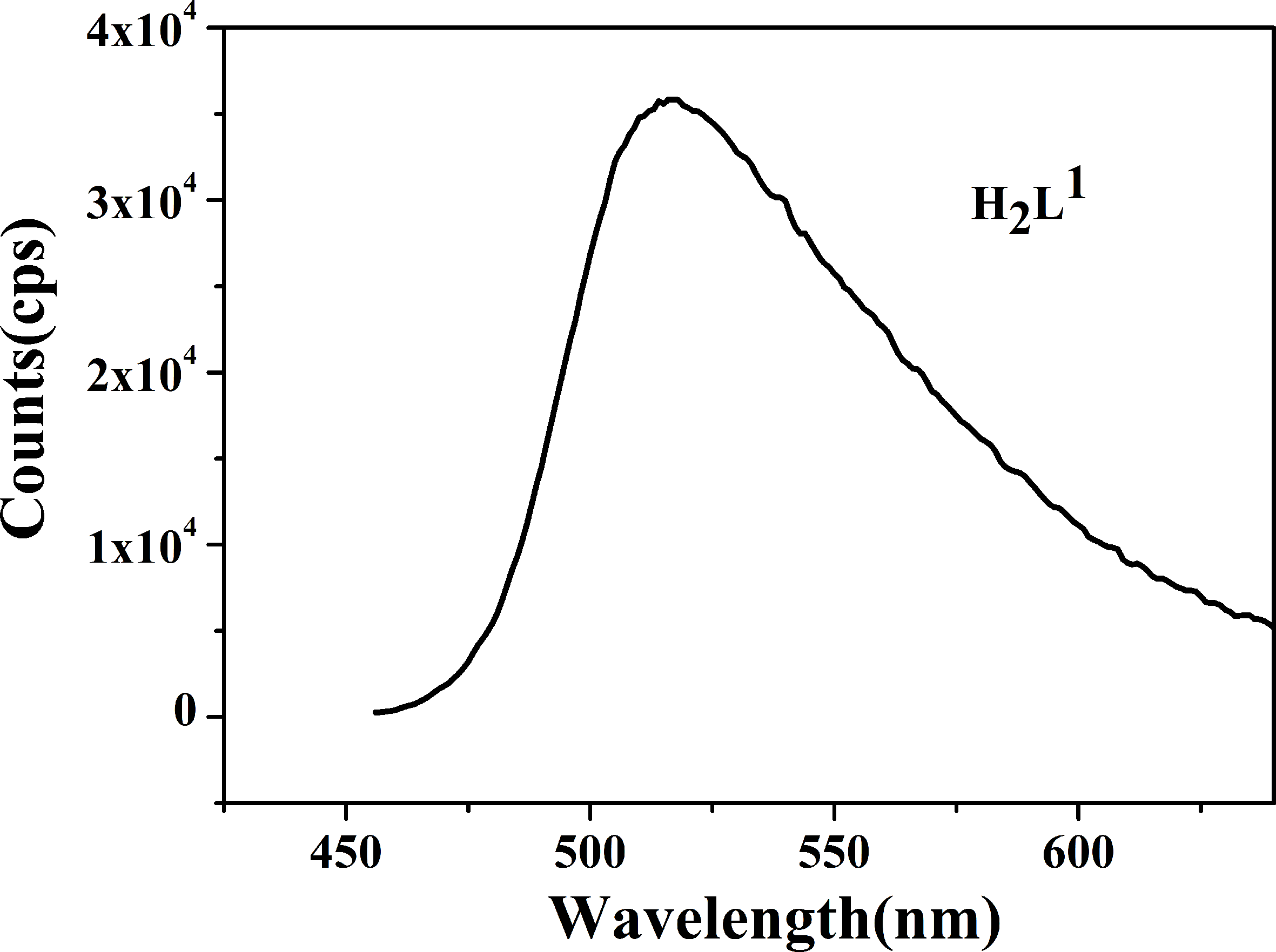


**S2 Fig. The solid–state emission spectra of H2L1 ligand at room temperature.**

Supplement: S2 Fig — (DOCX) [file pone.0130922.s002.docx]

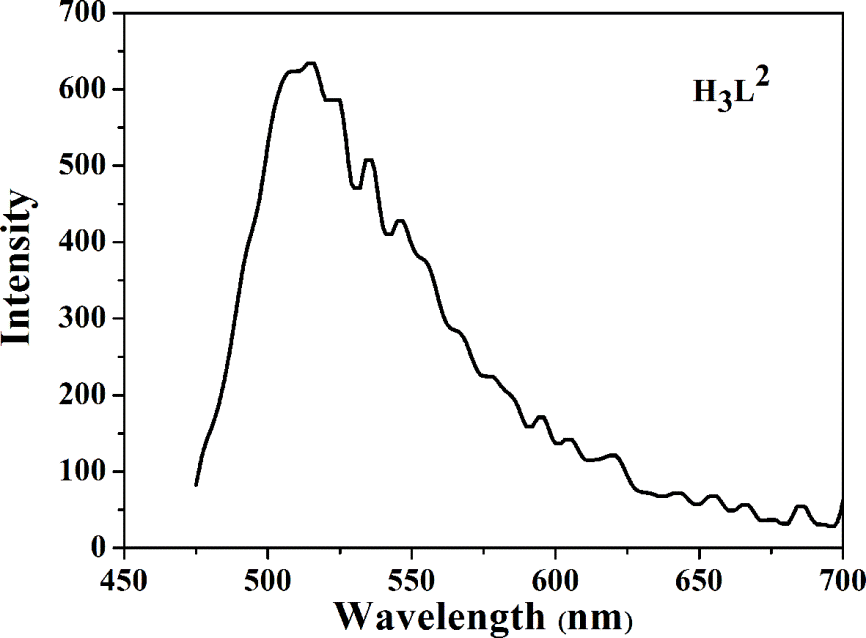


**S3 Fig. The solid–state emission spectra of** **H3L2 ligand at room temperature.**

Supplement: S3 Fig — (DOCX) [file pone.0130922.s003.docx]
