# Supplementary material for: Crystal Structure, Cytotoxicity and Interaction with DNA of Zinc (II) Complexes with o-Vanillin Schiff Base Ligands
Source: PLoS One. 2015 Jun 26;10(6):e0130922. doi: 10.1371/journal.pone.0130922 (PMC4482705; doi:10.1371/journal.pone.0130922)
Supplement: S1 Table — (DOCX) [file pone.0130922.s004.docx]

**S1 Table Selected bond lengths (Å) and bond angles (°) for complexes 1 and 2.**

| **Complex 1** | | | |
| --- | --- | --- | --- |
| Zn(1)-O(4) | 1.911(3) | Zn(1)-N(1) | 2.002(3) |
| Zn(1)-O(2) | 1.930(3) | Zn(1)-N(2) | 2.012(4) |
| O(4)-Zn(1)-O(2) | 111.59(16) | O(4)-Zn(1)-N(2) | 110.32(15) |
| O(4)-Zn(1)-N(1) | 96.05(14) | O(2)-Zn(1)-N(2) | 95.97(15) |
| O(2)-Zn(1)-N(1) | 113.44(16) | N(1)-Zn(1)-N(2) | 129.70(19) |
| **Complex 2** | | | |
| Zn(1)-O(4) | 1.900(11) | Zn(1)-N(1) | 1.992(12) |
| Zn(1)-O(5) | 1.986(10) | Zn(4)-N(2) | 2.024(10) |
| O(4)-Zn(1)-O(5) | 110.8(4) | O(1)-Zn(1)-O(10) | 165.1(4) |
| O(1)-Zn(2)-O(6) | 95.4(4) | O(4)-Zn(1)-N(1) | 123.0(5) |
| O(5)-Zn(1)-N(1) | 123.9(5) | O(18)-Zn(4)-N(2) | 115.9(5) |
